# Identifying cryptic population structure in multigenerational pedigrees in a Mexican American sample

**DOI:** 10.1186/1753-6561-8-S1-S4

**Published:** 2014-06-17

**Authors:** Robert C Culverhouse, Anthony L Hinrichs, Brian K Suarez

**Affiliations:** 1 Department of Medicine and Division of Biostatistics, Washington University School of Medicine, St. Louis, MO, 63110 USA; 2 Department of Psychiatry, Washington University School of Medicine, St. Louis, MO, 63110 USA; 3 Department of Genetics, Washington University School of Medicine, St. Louis, MO, 63110 USA

## Abstract

Cryptic population structure can increase both type I and type II errors. This is particularly problematic in case-control association studies of unrelated individuals. Some researchers believe that these problems are obviated in families. We argue here that this may not be the case, especially if families are drawn from a known admixed population such as Mexican Americans. We use a principal component approach to evaluate and visualize the results of three different approaches to searching for cryptic structure in the 20 multigenerational families of the Genetic Analysis Workshop 18 (GAW18). Approach 1 uses all family members in the sample to identify what might be considered "outlier" kindreds. Because families are likely to differ in size (in the GAW18 families, there is about a 4-fold difference in the number of typed individuals), approach 2 uses a weighting system that equalizes pedigree size. Approach 3 concentrates on the founders and the "marry-ins" because, in principle, the entire pedigree can be reconstructed with knowledge of the sequence of these unrelated individuals and genome-wide association study (GWAS) data on everyone else (to identify the position of recombinations). We demonstrate that these three approaches can yield very different insights about cryptic structure in a sample of families.

## Background

It is important for statistical geneticists to communicate with their colleagues that myriad preliminary analyses should be carried out before any formal analyses of the main hypotheses that motivated the study. Results of these preliminary analyses are crucial for making decisions about which phenotypic variables need to be conditioned on and which genotypes or individuals need to be dropped from the main analysis. These decisions need to be made before the formal analysis to keep the investigators from being influenced into making biased decisions supporting a particular hypothesis.

We believe that family studies of genome-wide sequence data, as well as studies based on unrelated individuals, should routinely examine their data for genetic heterogeneity. An early genome-wide linkage scan for prostate cancer illustrates why this could be of concern: half of the LOD score for the top genome-wide signal (1.4 out of 2.75) was due to just 2 out of the 91 families in the study. Those 2 families were African American, unlike the other 89 families, which were European American or Swedish [1]. This concern is heightened for analyses based on sequence data, where it is likely that causative variants may be found in a small subgroup or even in a single family [2]. In this paper, we present 3 ways to make such an initial evaluation using principal components (PCs) derived from a genome-wide screen. We illustrate these methods using the GAW18 data.

### The data

Mexican Americans are descendants of multiple ancestral populations, principally Native Americans, Europeans (primarily from the Iberian Peninsula) and Africans brought to the Americas as part of the slave trade [3]. We note that although this group is referred to as Latino or "Mexican"-Americans in the United States (because they historically have arrived in the US from Mexico), their Native American ancestry can be from Middle- or South-America as well as from the southern US and Mexico.

## Methods

### Data cleaning

Two sets of monozygotic twins were identified by the data providers. We dropped one monozygotic twin, at random, from each pair. We received these data after a cleaning algorithm had been applied by the data providers [4] but did not receive the original assessment of the quality of each call. We performed further cleaning to select the highest-quality markers for our principal component analysis (PCA). Complete details can be found in Hinrichs *et al*. [5]. Briefly, we identified markers with high call rates in both the GWAS data and sequencing data that were unambiguously mapped to the genome. We then pruned single-nucleotide polymorphisms (SNPs) to remove those in linkage disequilibrium (*r*^2 ^>0.5), which resulted in approximately 100,000 SNPs. We evaluated the resulting set of genotypes for Hardy-Weinberg equilibrium (HWE). The Q-Q plot did not reveal any deviations from expectation under the null. The final number of SNPs used here is 92,344.

### Outlier families

It has become common practice to analyze a GWAS sample of unrelated individuals for cryptic stratification, discarding the outliers. The definition of an outlier, however, is an unresolved issue in statistical analysis. Often, outliers are removed simply by visual inspection. Sometimes a more formal test is performed using, for instance, principles from numerical taxonomy. The question asked by this study is: Are all 20 pedigrees sufficiently homogeneous with regard to ancestry to be analyzed as a group with the same model parameters (e.g., gene frequencies)? Under approximate panmixia, we expect generational regression toward the group mean, especially in large pedigrees. Thus, in general, pedigrees offer more protection against outliers than a sample of unrelated individuals. It is well known, however, that immigrant groups are more likely to randomly mate *within *their own subgroups during the process of acculturation. Panmixia, with regard to the larger population, better describes the behavior of later generations. We used PCs [6] to determine the extent of clustering and whether any families can be considered outliers.

### Three approaches

Our goal was to evaluate structure within the sample of pedigrees rather than to estimate the ancestral contributions from Africans, Europeans, and Asians. To be sensitive to population substructures such as those known to exist in both European [7] and Native American populations [8], we focused on unsupervised Eigenstrat analyses [6], including only the sample data.

Given this decision, there remain multiple reasonable ways to derive PCs for the data that address the correlation within the pedigrees. We examined 3 such approaches. First, we used all the data, ignoring pedigree membership. This represents the diversity of the data as a whole but may be distorted by differing pedigree sizes. In the GAW18 data, the smallest genotyped family contained 22 individuals, and the largest consisted of 86 genotyped individuals. The second approach also preserves allele frequencies within families but weights individuals proportionally to the inverse of the pedigree size so that the families contribute equally to the determination of PCs. The third approach concentrates on the set of maximally unrelated individuals. The motivation for this approach is that, in principle, the sequence of all family members can be reconstructed from the sequence of the founders and marry-ins. Dense (and relatively inexpensive) SNP data on the remaining unsequenced members (to allow accurate inference of the location of each meiotic recombination event) can then be used to reconstruct the genotypes of the entire kindred.

Each of these approaches can give insight into the ancestral structure of pedigrees in a family-based study. We examined the resulting PCs from each of these approaches for the GAW18 families. Because the GAW18 data were not simulated with population substructure in mind, we did not attempt to correlate the differences we found to differences in phenotypes.

## Results

### Approach 1: Principal components based on the original sample

**Figure 1:**
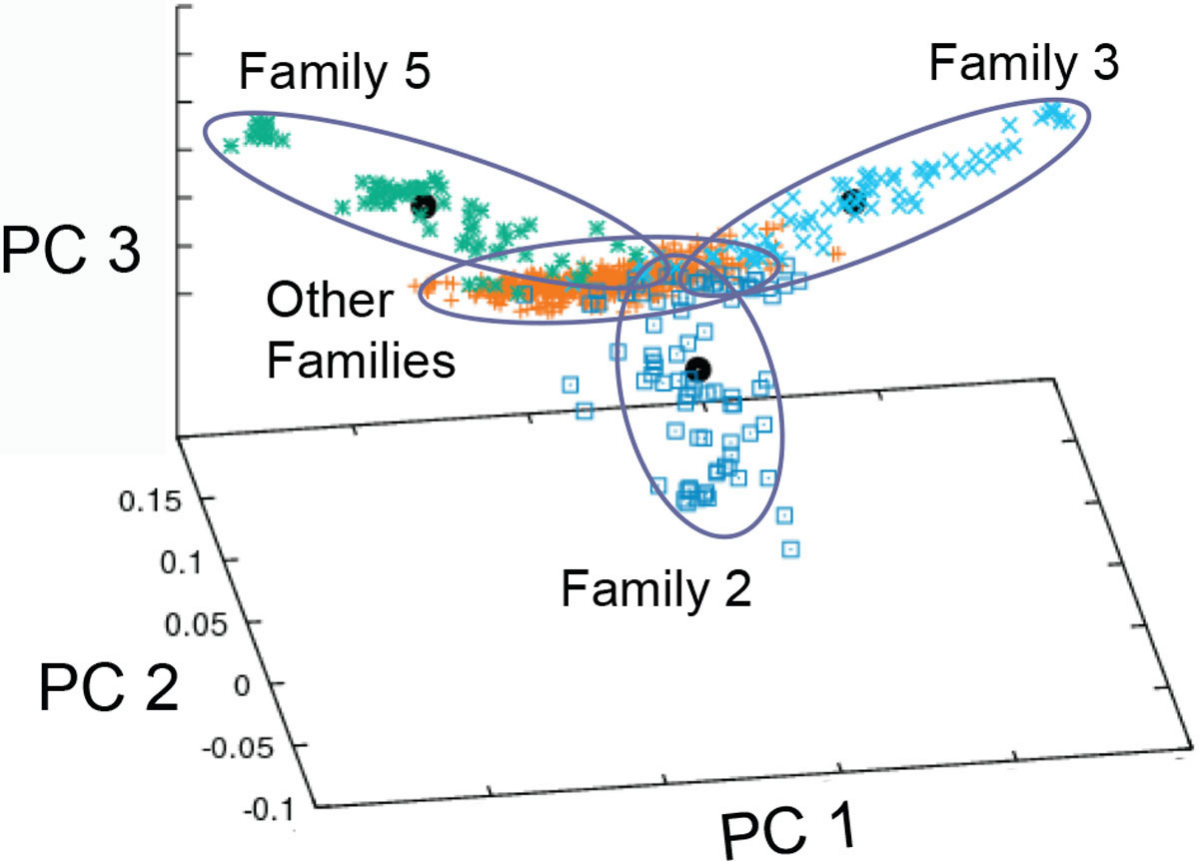
****Individual and family centroids using principal components (PCs) based on all subjects****. Eigenstrat analyses of the first three PCs of all members of the 20 large pedigrees. The heavy black dots denote the centroid of each "outlier" family. The members of the remaining 17 families are in orange.

An examination of the eigenvalues for the PCs (scree plot) suggested that the first 3 PCs contained the most useful information for these data. They accounted for 1.37%, 1.04%, and 0.98% of the variance, respectively, for a total of 3.39% of variance. (For comparison, the first two PCs from an analysis of genetic clinical variation in Europeans account for 0.30% and 0.15%. [9].) Figure 1 shows the distribution of the individuals in the 20 pedigrees for the first 3 PCs. The centroid is, of course, at the origin of the eigenvectors. There are two easily discernible tails: one trailing off toward the upper left quadrant (family 5) and one trailing off to the upper right quadrant (family 3). A third family, differentiated primarily by the third PC, can be seen dropping below the plane defined by the first 2 PCs (family 2). We removed each of the 20 families in turn, recomputed the centroid and standard deviation distances for the remaining data, and compared the centroid of the excluded family to the remaining data. This information is summarized in Table 1. Clearly, under this metric, each of these 3 families differs from the remaining families.

**Table 1:** Distance from family centroids to centroid of remaining data

Family	# of SD to center of the rest of the data
2	2.4
3	2.5
5	3.0
All others	0.1 to 0.8

For each family, the unit SD metric is defined by the standard deviation of all the distances from individuals not in that family to the centroid of all individuals not in that family.

### Approach 2: Principal components based on the proportionally weighted families

**Figure 2:**
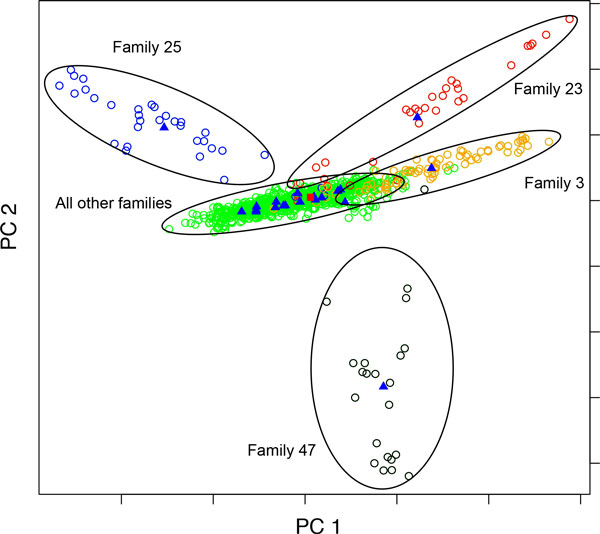
**Individuals and family centroids using principal components (PCs) based on equally weighted families**. Individuals are represented by circles, family centroids by blue triangles, and the global centroid by a red square.

Because the genotypes of pedigree members are correlated and families differ in size, there is a danger that large families could "swamp out" variation in the smaller pedigrees. Accordingly, we reweighted our sample so each pedigree would have the same effective sample size. Examination of the scree plot for these PCs suggests that the first two PCs are informative. They account for 1.26% and 1.13% of the variance individually and 2.39% together. Figure 2 reveals a very different picture compared with Figure 1. We anticipated this because families 2, 3, and 5, highlighted in Figure 1, are among the largest (*N *= 86, 76, and 68, respectively). The remaining families range in size from 22 to 68 (median family size = 41.5; mean family size = 47.85). Figure 2 shows the first two PCs when the weighting approach is used. Family 3 can still be distinguished (its centroid lies outside those of the other 16 families), but now families 23, 25, and 47 appear to be outliers. These three families are the smallest for which we have genotype data.

### Approach 3: Principal components based on a maximal set of unrelated individuals

**Figure 3:**
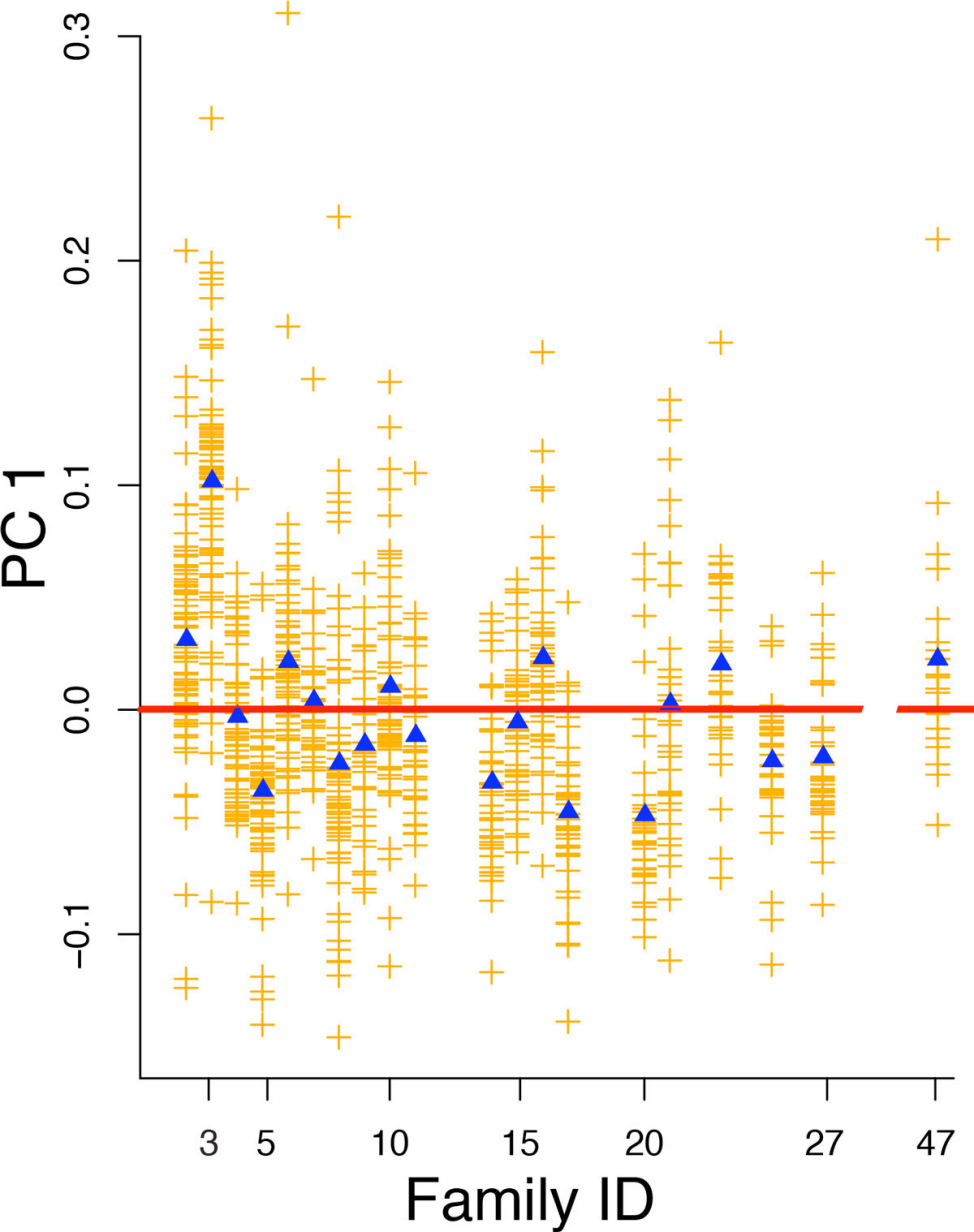
**Individual and family means of first principal components (PCs) derived from a set of maximally unrelated individuals**. Individuals are represented by orange +s, family means by blue triangles, and the overall mean by the red line at 0. Each nonempty column represents the family whose ID is listed on the x-axis.

Approach 3 uses just the founders and the marry-ins. Examination of the scree plot for these PCs suggests that only the first PC is informative, accounting for 1.64% of the variance. Figure 3 reports the results of this analysis. This plot is unremarkable but clearly distinguishes family 3 from the others.

## Discussion

It is well known that the presence of unrecognized stratification can lead to an increase in type I or type II errors in linkage or association analyses when model parameters are misspecified. When confronted with heterogeneity, an investigator interested in performing a linkage analysis has at least two choices. First, homogeneous subsets of the data can be analyzed separately and the resulting statistics combined. A second option is available with most linkage programs. This option requires the recoding of alleles in one subgroup (with frequency estimates appropriate to that group). This tedious procedure allows the entire sample of families to be analyzed together [10].

**Figure 4:**
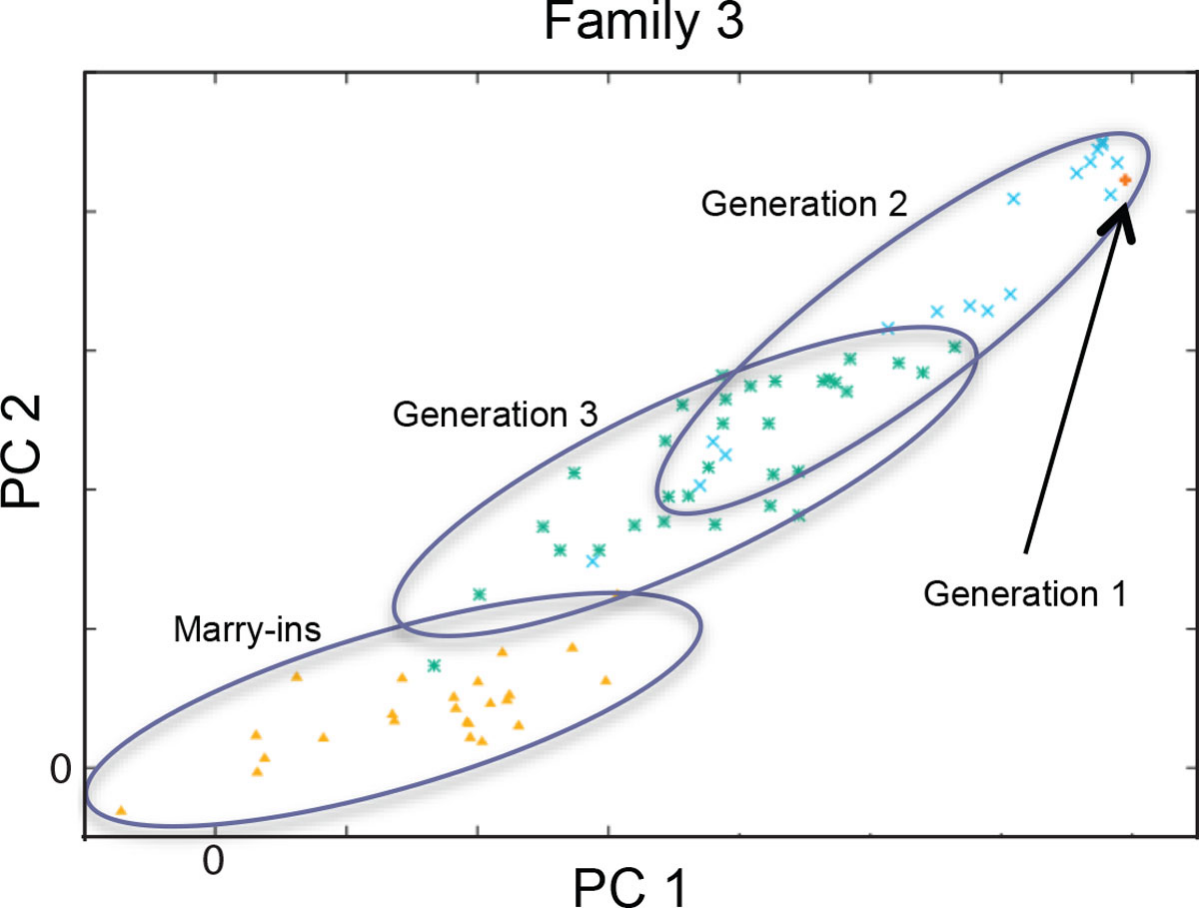
**Generational diagram for family 3 based on the first two principal components (PCs) from approach 1**. Family 3 demonstrates a generational "regression to the mean." The marry-ins are genetically indistinguishable from the central mass of the other families. As a result, each subsequent generation is more similar to the main body of the GAW18 sample.

As mentioned earlier, outlier families undergoing acculturation usually show regression to the larger group mean. Family 3 illustrates this phenomenon (Figure 4). Only one child of the founders of this family was genotyped (denoted by a + in the upper right quadrant). The unrelated spouses who married in the pedigree tend to be close to the origin, and the founders' grandchildren and great-grandchildren tend to be located near the midpoint of the parents, as expected. Within a few generations, we expect this family (and family 5, not shown) to display genetic variation similar to that of the other Mexican American families in this sample.

When comparing the results from our approaches to a supervised principal component derivation using the YRI, CEU, and CHB+JPT population samples from HapMap, we notice that the oldest member of pedigree 3 lies in the CEU cluster, unlike members of the other families. Because this individual had many descendants, "more European" may explain why pedigree 3 is identified as an outlier by all 3 approaches. It is less clear what history distinguishes families 2 and 5 from the rest. It is possible, although we do not have data to be certain, that their differences relate to substructure within their Native American ancestry (e.g., Zapotec vs. Tlaxcalan).

## Conclusions

Family-based methods generally are not immune to difficulties related to cryptic population structure (although some methods, such as the TDT, are). We believe it is important to include an investigation of the potential differences among families at the beginning of analyses, similar to the methods used to identify outlier individuals. Possible responses to the detection of substructure range from removing a family from the analysis to using PCs as adjustment covariates in the analysis or simply using this information when interpreting results from an association test. If an association between a phenotype and a variant is primarily due to a single pedigree (as was found in the GAW17 data), understanding the cryptic structure of the data under one or more of these metrics may prove useful for interpreting the results.

## Competing interests

The authors declare that they have no competing interests.

## Authors' contributions

RCC, ALH, and BKS designed the overall study. RCC and ALH conducted statistical analyses; and RCC and BKS drafted the manuscript. All authors read and approved the final manuscript.
